# Selection of suitable reference genes for quantitative real-time polymerase chain reaction in human meningiomas and arachnoidea

**DOI:** 10.1186/1756-0500-4-275

**Published:** 2011-08-02

**Authors:** Christina Pfister, Marcos S Tatabiga, Florian Roser

**Affiliations:** 1 Department of Neurosurgery, University of Tuebingen, Hoppe-Seyler-Str.3, 72076 Tuebingen, Germany

## Abstract

**Findings:**

At first 32 housekeeping genes were analyzed in six randomly chosen meningiomas, brain and dura mater using geNorm, NormFinder, Bestkeeper-1 software and the comparative ΔCt method. Reference genes were ranked according to an integration tool for analyzing reference genes expression based on those four algorithms. Eight highest ranked reference genes (CASC3, EIF2B1, IPO8, MRPL19, PGK1, POP4, PPIA, and RPL37A) plus GAPDH and ACTB were then analyzed in 35 meningiomas, arachnoidea, dura mater and normal brain. NormFinder and Bestkeeper-1 identified RPL37A as the most stable expressed gene in meningiomas and their normal control tissue. NormFinder also determined the best combination of genes: RPL37A and EIF2B1. Commonly used reference genes GAPDH and ACTB were considered least stable genes. The critical influence of reference genes on qPCR data analysis is shown for VEGFA transcription patterns.

**Background:**

In meningiomas quantitative real-time reverse transcription-polymerase chain reaction (qPCR) is most frequently used for accurate determination of gene expression using various reference genes. Although meningiomas are a heterogeneous group of tissue, no data have been reported to validate reference genes for meningiomas and their control tissues.

**Conclusions:**

RPL37A is the optimal single reference gene for normalization of gene expression in meningiomas and their control tissues, although the use of the combination of RPL37A and EIF2B1 would provide more stable results.

## Background

Meningiomas are the most frequent intracranial tumours. They originate from the arachnoidal cap cells of the meningeal coverings of the spinal cord and brain, constituting for approximatively 13 to 26% of all intracranial pathologies [1,2]. The conventional strategy for meningiomas is surgery [3,4]. However, some meningiomas recur as resection might be sub totally due to their delicate location at skull-based structures. The definition of malignant potential is beset by the frequent discordance between histology and biology [5,6]. Meningiomas are categorized in three WHO grades, in which there are several subtypes differentiated by their histological features.

Real-time quantitative reverse transcription polymerase chain reaction (RT-qPCR) is a sensitive and reliable method for quantifying gene expression. Most frequently the relative quantification method is used, which requires the use of an internal control gene for normalization. Reference genes are mostly genes, which are involved in basic metabolism and maintenance of the cell. An ideal reference gene should be expressed at a constant level in all examined tissues and cells, and should not be influenced by experimental conditions. However several studies have shown, that genes used as reference gene display significantly different gene expression levels [7-9].

Established housekeeping genes in meningioma RT-qPCR experiments are genes such as glyceraldehyde-3-phosphate dehydrogenase (GAPDH) and β-Actin (ACTB) [10-18] as well as ribosomal RNA (18S rRNA) and TATA binding box protein (TBP) [19-21]. As the application of these various housekeeping genes shows, there are no reports that candidate reference genes in meningiomas were validated. Due to the heterogeneity of meningioma tissue and the necessity to compare meningiomas and their control tissue reliably, the selection of an appropriate reference gene with stable gene expression throughout the various tissues is essential for further use of RT-qPCR in meningioma research.

In this study, we investigate the gene expression levels of 32 commonly used housekeeping genes in meningiomas and their control tissues arachnoidea, dura and normal brain. The RT-qPCR results were analyzed with four different algorithms, to select eight suitable reference genes. Those genes plus GAPDH and ACTB were compared in an increased number of meningiomas and control tissues. These RT-qPCR results were further analyzed with two different algorithms: NormFinder and Bestkeeper-1.

## Methods

### Tumour Specimens and Cell Culture

Meningioma surgical specimens as well as arachnoidea and dura mater were obtained from the Neurosurgical Department in accordance to regulations of the Ethic Committee of the University of Tuebingen. Primary cultures were obtained from tumour tissue samples within 30 minutes of surgical removal. Samples were first washed in phosphate-buffered saline (PBS), reduced and mashed through a filter and placed in Dulbecco's modified Eagle's medium (DMEM) with fetal bovine serum (FBS), 2 mmol/L L-glutamine and 0,1% 10 mg/ml Gentamicin (Invitrogen, Grand Island, NY). Cells were plated in 25-mm^2 ^tissue culture flasks and incubated at 37°C in a humidified atmosphere of 5% CO_2_. Medium was changed every 3 to 4 days and cultures were split using 600 μl Accutase (PAA, Pasching; Austria). Viable cells were stored in liquid nitrogen in 90% medium/10% dimethyl sulfoxide.

### RNA isolation and reverse transcription

Meningioma total ribonucleic acid (RNA) was isolated directly from primary cell cultures before splitting and RNA of Arachnoidea and dura was isolated from fresh tissue using PARIS^® ^kit (Ambion, Inc., Austin, TX), according to the manufacturer's protocol. RNA was treated with DNA-free™ (Ambion, Inc., Austin, TX) to remove residual genomic DNA. The concentration of the isolated RNA and the 260/280 absorbance ratio was measured in triplicates with Eppendorf Biophotometer (Eppendorf, Hamburg, Germany). The integrity of RNA samples was confirmed by electrophoresis on a 2% Sybr Green agarose gel (Invitrogen Inc., Carlsbad, CA). The criterion to include RNA samples was 260/280 ~ 2 (1.9 to 2.2) and 28S/18S ratio ≥ 1.7. The probes were stored at - 80°C until use. For normal brain FirstChoice^® ^Human Brain Reference RNA (Ambion, Inc., Austin, TX) was used, which pools RNA from different donors and several brain regions. RNA samples were DNase treated using DNAfree kit (Ambion Inc., Austin, TX). Total RNA (1 μg) was reverse-transcribed to cDNA using des High Capacity RNA-to-cDNA Kits (Applied Biosystems, Foster City, CA) in a total volume of 20 μl, according to the manufacturer's protocol.

### Primer selection

For investigation to identify the most stable reference gene that could be used for normalization in RT-qPCR studies in meningiomas TaqMan^® ^Express Plate Human Endogenous Control Plates (Applied Biosystems, Foster City, CA) were used, which contained 32 different genes plated in triplicates (Table 1). This collection of genes has been selected from literature searches and/or whole genome microarray tests carried out on numerous human tissues. They have been shown to be expressed constitutively and at moderate abundance across most test samples. All primers further evaluated spanned an exon junction to minimize inaccuracies due to genomic DNA contamination in RNA samples except the primer for S18. Additional information on assay optimisation and validation such as primer sequence for each TaqMan^® ^Assay are available from Applied Biosystems. Priming conditions, primer concentration and annealing temperature was identical in all used TaqMan^® ^Gene Expression Assays. TaqMan^®^-based detection was chosen, because this detection method detects only specific amplification products, whereas SYBR^®^-Green based detection detects all amplified double-stranded DNA, including nonspecific double-stranded DNA sequences, which may generate false positive signals. Melt curves were not assessed because they are only suitable for SYBR^®^-Green based detection.

**Table 1:** Candidate reference genes evaluated in this study

Gene Symbol	Gene Name	**Genbank Acession No**.	**TaqMan**^**®**^**Assay ID**	Amplicon length
18S	Eukaryotic 18S rRNA	X03205.1 (mRNA)	Hs99999901_s1	187
ABL1	v-abl Abelson murine leukemia oncogene homolog 1	NM_005157.3 + NM_007313.2	Hs00245445_m1	91
**ACTB**	**Actin, Beta, cytoplasmic**	NM_001101.3	**Hs99999903_m1**	**171**
B2M	Beta-2-microglobulin	NM_004048.2	Hs99999907_m1	75
**CASC3**	**cancer susceptibility candidate 3**	NM_007359.4	**Hs00201226_m1**	**67**
CDKN1A	cyclin-dependent kinase inhibitor 1A (p21, Cip1)	NM_078467.1 + NM_000389.3	Hs00355782_m1	66
CDKN1B	cyclin-dependent kinase inhibitor 1B (p27, Kip1)	NM_004064.3	Hs00153277_m1	71
**EIF2B1**	**eukaryotic translation initiation factor 2B, subunit 1 alpha, 26 kDa**	NM_001414.3	**Hs00426752_m1**	**75**
ELF1	E74-like factor 1/ets domain transcription factor)	NM_172373.3 + NM_001145353.1	Hs00152844_m1	76
GADD45A	growth arrest and DNA-damage-inducible, alpha	NM_001924.2	Hs00169255_m1	123
**GAPDH**	**Glyceraldehyde-3-phosphate dehydrogenase**	NM_002046.3	**Hs99999905_m1**	**122**
GUSB	Glucuronidase, Beta	NM_000181.3	Hs99999908_m1	81
HMBS	Hydromethylbilane synthase	NM_000190.3	Hs00609297_m1	64
HPRT1	Hypoxanthine guanine phospho- ribosyl transferase 1	NM_000194.2	Hs99999909_m1	100
**IPO8**	**Improtin 8**	NM_006390.2	**Hs00183553_m1**	**71**
**MRPL19**	**mitochondrial ribosomal protein L19**	NM_014763.3	**Hs00608519_m1**	**72**
MT-ATP6	mitochondrially encoded ATP synthase 6	NC_001807.ATP6.0	Hs02596862_g1	150
PES1	pescadillo homolog 1, containing BRCT domain (zebrafish)	NM_014303.2	Hs00362795_g1	56
**PGK1**	**Phosphoglycerate kinase 1**	NM_000291.3	**Hs99999906_m1**	**75**
POLR2A	Polymerase (RNA) II (DNA directed) polypeptide A, 220 kDa	NM_000937.3	Hs00172181_m1	61
**POP4**	**processing of precursor 4, ribonuclease P/MRP subunit**	NM_006627.2	**Hs00198357_m1**	**68**
**PPIA**	**Peptidylprolyl Isomerase A**	NM_021130.3	**Hs99999904_m1**	**98**
PSMC4	proteasome (prosome, macropain) 26S subunit, ATPase, 4	NM_153001.1 + NM_006503.2	Hs00197826_m1	83
PUM1	pumilio homolog 1 (Drosophila)	NM_001020658.1 + NM_014676.2	Hs00206469_m1	89
RPL30	ribosomal protein L30	NM_000989.2	Hs00265497_m1	149
**RPL37A**	**ribosomal protein L37A**	NM_000998.4	**Hs01102345_m1**	**125**
RPLP0	Ribosomal protein, large, P0	NM_053275.3 + NM_001002.3	Hs99999902_m1	105
RPS17	ribosomal protein S17	NM_001021.3	Hs00734303_g1	93
TBP	TATA binding box protein	NM_008907	Hs99999910_m1	127
TFRC	Transferrin receptor	NM_001128148.1 + NM_003234.2	Hs99999911_m1	105
UBC	Ubiquitin C	NM_021009.4	Hs00824723_m1	71
YWHAZ	Tyrosine 3-monooxygenase	NM_003406.3	Hs00237047_m1	70

Thickly printed reference genes were further evaluated. TaqMan^® ^Assay ID ending with "_m" indicates an assay whose probe spans an exon junction and will not detect genomic DNA. "_g" indicates an assay that may detect genomic DNA. The assay primers and probe may also be within a single exon. "_s" indicates an assay whose primers and probes are designed within a single exon, such assays will, by definition detect genomic DNA. Additional information for each TaqMan^® ^Assay is available from Applied Biosystems.

For further evaluation single TaqMan^® ^Gene Expression Assays for ACTB, CASC3, EIF2B1, GAPDH, IPO8, MRPL19, PGK1, POP4, PPIA, RPL37A (Applied Biosystems, Foster City, CA) were used, which were identical with the assays used in TaqMan^® ^Express Plate Human Endogenous Control Plates.

### Real-time PCR

TaqMan^® ^real-time PCR was run in triplicates in 48-well reaction plates with a StepOne™ (Applied Biosystems, Foster City, CA). Real-time PCR reaction was performed with 1 μl cDNA (5 ng/μl) in 20 μl reaction mix containing 10 μl TaqMan^® ^Gene Expression Master Mix (Applied Biosystems, Foster City, CA) and 1 μl TaqMan^® ^Gene Expression Assays (Applied Biosystems, Foster City, CA). The cycling conditions were as follows: initial holding period at 95°C for 10 min, followed by a two-step PCR program consisting of 95°C for 15 s and 60°C for 1 min for 40 cycles. Reverse transcriptase negative controls and "no template controls" (without cDNA in PCR) were included. Data were collected and quantitatively analyzed using StepOne™ Software v2.1. Relative quantitation analysis of gene expression data for VEGFA analysis was conducted according to the 2^-ΔΔCt ^method [22].

For PCR efficiency a 5-fold dilution series was created from a random pool of cDNA from our sample group ranging from 50 ng to 0.08 ng. PCR were performed as described above in triplicate. The PCR efficiency and correlation coefficients (R^2^) of each TaqMan^® ^Gene Expression Assay were generated using the slops of the standard curves. The efficiencies were calculated by the formula: efficiency (%) = (10^(-1/slope) ^-1) * 100. All assays displayed efficiencies between 93.2% and 100.2% (Table 2).

**Table 2:** Efficiency data for evaluated genes

Gene symbol	Slope	**R**^**2**^	Efficiency (100%)
ACTB	- 3.420	1.000	96.1
CASC3	- 3.442	0.999	95.2
EIF2B1	- 3.434	0.997	95.5
GAPDH	- 3.430	1.000	95.7
IPO8	- 3.390	1.00	97.3
MRPL19	- 3.364	0.999	98.3
PGK1	- 3.266	0.994	100.2
POP4	- 3.410	0.999	96.4
PPIA	- 3.497	1.00	93.2
RPL37A	- 3.406	1.000	96.6

### Statistical analysis

To compare the stability of candidate reference genes, four validation software programs were used according to their original publication: geNorm http://medgen.ugent.be/~jvdesomp/genorm[23], NormFinder http://www.mdl.dk/publicationsnormfinder.htm[24], BestKeeper-1 http://www.gene-quantification.de/bestkeeper.html[25] and the comparative delta Ct method [26]. For geNorm and NormFinder the raw C_t _values were transformed to quantities by using the delta C_t _method [27]. The highest relative quantities for each gene were set to 1. Bestkeeper-1 and the comparative delta Ct method use raw C_t _values. To evaluate the results from the four algorithms an integration tool for analyzing reference genes expression was used http://www.leonxie.com/referencegene.php. First according to the reference genes ranking by every algorithm from the most stable gene to the least stable gene, a series of continuous integers starting from 1 as weight to each reference gene is assigned. The geomean of each gene weights across the four algorithms is calculated and then these reference genes are re-ranked. The gene with the less geomean is viewed as more stable reference gene. Input data is value data from Real-Time qRT-PCR. Statistical analysis was performed with GraphPad Prism V5.03 (GraphPad Software, La Jolla, USA). Normality was assessed according to D'Agostino-Pearson tests with alpha = 0.05. For evaluation of statistical equivalence a confidence-interval version of the Two One-Sided Tests (TOST) procedure of Schuirmann was used [28]. The groups are considered equivalent at a 5% significance level if their difference has a 90% confidence interval that lies entirely inside the upper and lower equivalence limits. Therefore we considered ± δ = ± 1.5 to be reasonable limits of equivalence.

## Results

### Expression levels of 32 reference genes in meningioma and normal tissue

**Figure 1:**
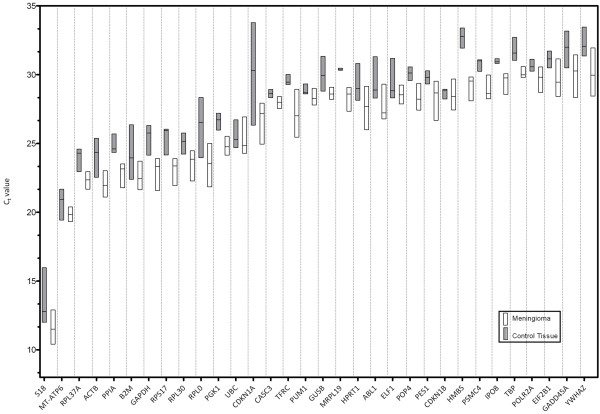
**Expression levels of 32 candidate reference genes**. Expression levels of 32 reference genes in four meningiomas, the malignant meningioma cell line IOMM-Lee, normal brain, cerebral meninges and dura mater. Real-Time PCR cycle threshold numbers are shown (C_t _value). Box plots represent maximum and minimum values with median. Lower C_t _values indicate higher gene expression.

To select suitable reference genes TaqMan^® ^human endogenous control plates (Applied Biosystems, Foster City, CA, USA) were used containing 32 known housekeeping genes (Table 1). Four different meningiomas, the malignant meningioma cell line IOMM-Lee, normal brain, cerebral meninges and dura mater were analyzed regarding the gene expression levels of those housekeeping genes. The mean C_t _values displayed a wide range of expression levels between 10.41 and 33.78 as shown in Figure 1. The most abundant transcript was S18 with median C_t _value of 11.50 in meningiomas and a mean C_t _value of 13.59 in normal tissue. In meningioma the lowest expressed genes were YHWAZ with median C_t _value of 30.27 and TBP with 30.08. In normal tissue HMBS had the lowest expression with a median C_t _value of 32.70. Tumour tissue and normal tissue group of five candidate reference genes (CASC3, CDKN1B, POLR2A, PUM1 and UBC) were statistically equivalent to within ± 1.5.

### Expression stability of 32 candidate reference genes in meningioma and brain

**Figure 2:**
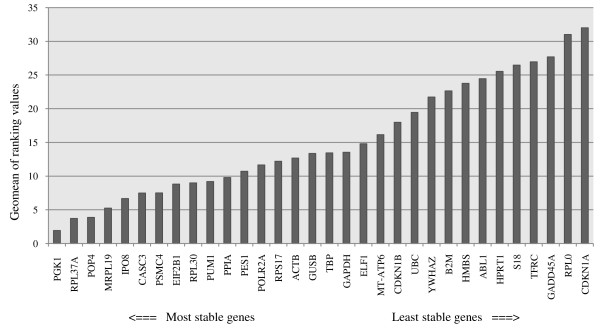
**Comprehensive gene stability of 32 candidate reference genes in meningiomas and control tissue**. Geomean of ranking values (y-axis) of 32 reference genes with their ranking from least to most stable gene expression (x-axis). Lower geomean values indicate more stable gene expression.

All 32 housekeeping genes were analyzed using four different algorithms, geNorm NormFinder, Bestkeeper-2 and the comparative delta Ct method. An integration tool calculated the geomean of each gene across the four algorithms and ranked the reference genes according to their comprehensive gene stability (Figure 2). The three most stable reference genes were PGK1 > RPL37A > POP4. The least stable reference genes were CDKN1A > RPL0 > GADD45A. Three of four used algorithms ranked PGK1 highest, only Bestkeeper-1 ranked CASC3 highest and PGK1 only in fourteenth place. For further analysis eight of the highest ranked expression genes were chosen: PGK1, RPL37A, POP4, MRPL19, IPO8 and CASC3. Additionally the most used reference genes in meningioma qPCR experiments ACTB and GAPDH were also chosen although being only ranked in fifteenth respectively eighteens place and being considered inconsistent with a standard deviation (SD) higher than 1 by Bestkeeper-1. Three reference genes (CDKN1B, UBC and POLR2A) with equivalent tumour and normal tissue group were ranked low from position 19 to 22 respectively 13. CASC3 was the only one included for further investigation with statistically equal groups.

### Expression stability of eight reference genes plus GAPDH and ACTB in meningiomas, arachnoidea, dura and normal brain

To validate the expression stability of CASC3, EIF2B1, IPO8, MRPL19, PGK1, POP4, PPIA, RPL37A plus GAPDH and ACTB, thirty-four randomly chosen primary cultured meningiomas, the meningioma cell line IOMM-Lee, two arachnoidea, six dura mater, one cerebral meninges and two pooled normal brain samples were screened for these reference genes. For analysis two different algorithms were chosen: NormFinder and Bestkeeper-1. NormFinder has a model-based approach whereas Bestkeeper-1 employs a pair-wise correlation analysis. NormFinder also estimates the variation between subgroups such as normal and cancer tissue.

Both algorithms identified RPL37A as the most stable gene in meningiomas and normal control tissue with an average expression stability value (M) value of 0.54 (Bestkeeper-1) respectively 0.12 (NormFinder). NormFinder not only determines the most stable gene, but also the best combination of two genes, which are RPL73A and EIF2B1 with a stability value of 0.088. The remaining ranking differed significantly for Bestkeeper-1 and NormFinder (Table 3). Bestkeeper-1 considered ACTB inconsistent with SD = 1.00 in meningiomas and their control tissue, whereas PGK1 was inconsistent in meningiomas (SD = 1.04). Both algorithms determined EIF2B1 and RPL37A as the two most stable genes in normal tissue (Table 4). In contrast there were significant differences between the ranking of Bestkeeper-1 and NormFinder in meningiomas (Table 5). Bestkeeper-1 ranked CASC3 and RPL37A highest. NormFinder identified MRPL19 and POP4 as the two most suitable genes, ranking CASC3 and RPL37A in eighth respectively ninth position.

**Table 3:** Ranking of ten candidate reference genes in meningiomas and their control tissue based on average expression stability value as calculated by Bestkeeper-1 and NormFinder.

Rank	Bestkeeper-1		NormFinder	
		
	Gene name	Stability value	Gene name	Stability value
1	RPL37A	0.54	RPL37A	0.118
2	CASC3	0.54	EIF2B1	0.122
3	MRPL19	0.65	POP4	0.155
4	IPO8	0.66	MRPL19	0.163
5	POP4	0.70	PGK1	0.169
6	PPIA	0.73	PPIA	0.200
7	EIF2B1	0.74	GAPDH	0.286
8	PGK1	0.76	ACTB	0.287
9	GAPDH	0.83	CASC3	0.289
10	ACTB	1.00	IPO8	0.380

**Table 4:** Ranking of ten candidate reference genes in normal control tissue based on average expression stability value as calculated by Bestkeeper-1 and NormFinder.

Rank	Bestkeeper-1		NormFinder	
		
	Gene name	Stability value	Gene name	Stability value
1	EIF2B1	0.45	EIF2B1	0.306
2	RPL37A	0.56	RPL37A	0.318
3	PPIA	0.65	MRPL19	0.329
4	MRPL19	0.69	PPIA	0.404
5	ACTB	0.75	GAPDH	0.415
6	POP4	0.77	POP4	0.419
7	IPO8	0.82	CASC3	0.462
8	GAPDH	0.82	ACTB	0.542
9	CASC3	0.85	PGK1	0.590
10	PGK1	1.04	IPO8	0.621

**Table 5:** Ranking of ten candidate reference genes in meningiomas based on average expression stability value as calculated by Bestkeeper-1 and NormFinder.

Rank	Bestkeeper-1		NormFinder	
		
	Gene name	Stability value	Gene name	Stability value
1	CASC3	0.43	MRPL19	0.197
2	RPL37A	0.45	POP4	0.269
3	MRPL19	0.59	IPO8	0.277
4	IPO8	0.62	PPIA	0.312
5	PPIA	0.63	PGK1	0.332
6	POP4	0.63	EIF2B1	0.355
7	PGK1	0.67	GAPDH	0.386
8	EIF2B1	0.70	CASC3	0.388
9	GAPDH	0.75	RPL37A	0.447
10	ACTB	0.95	ACTB	0.521

TOST procedure showed statistical equivalence between normal tissue and meningiomas (± δ = ± 1.5) for three reference genes: CASC3 (+0.87), IPO8 (+0.57) and POP4 (+1.36). Those three genes were not normally distributed in meningiomas (CASC3 (P-value = 0.002), IPO8 (P-value < 0.0001) and POP4 (P-value = 0.0005). After inclusion of the normal tissue group IPO8 and POP4 remained not normally distributed.

### Contribution of reference genes on expression levels of target genes

**Figure 3:**
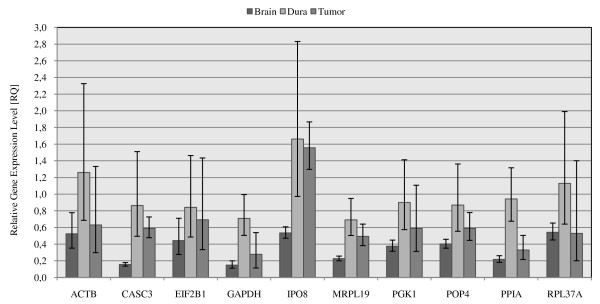
**Contribution of reference gene selection to determination of target gene expression levels**. Data is expressed as relative gene expression levels (RQ) to arachnoidea (RQ_arachnoidea _= 1). RQ_min. _and RQ_max. _are calculated with a confidence level of 95%.

The selection of a reference gene for normalisation of qPCR can have a distinct influence on the expression profile of target genes [29]. To show the influence of different reference genes on the determination of gene expression levels, VEGFA expression levels in meningiomas and their control tissues were sequentially normalized with the analyzed ten reference genes. The C_t _values for VEGFA were between 29 and 31. The expression level of VEGFA was normalized to each single reference gene as shown in Figure 3. The relative gene expression level (RQ) of VEGFA was calculated relative to the arachnoidea group (RQ_Arachnoidea _= 1). Subsequent bars represented the different expression levels of VEGFA in normal brain, dura and meningiomas normalized by different reference genes. Most reference genes maintained the ratio between brain, dura and meningioma except IPO8 and CASC3. Normalization with IPO8 or CASC3 showed significantly increased ratio for brain to dura and brain to tumour.

## Discussion

The requirement for distinct and reproducible results from quantitative gene expression analysis is accurate data normalization [23,24,29,30]. The application of an inappropriate reference gene can lead to false experimental conclusions [31-33]. Therefore one or more reference need to be chosen dependent on used tissue and experimental conditions.

To our knowledge, this is the first systematic analysis of average expression stability of reference genes in meningiomas for data normalisation in qPCR experiments. To evaluate the average expression stability four analysis software programs (geNorm, NormFinder, Bestkeeper-1 and the comparative delta Ct method) based on different algorithms were used. So far various reference genes (GAPDH, ACTB, S18, TBP) were used in qPCR experiments in meningiomas [11-21,34], although GAPDH was mainly used for normalizations. This study demonstrates that none of these reference genes were ranked under the ten most stable genes of 32 analyzed reference genes. However GAPDH and ACTB as the most used reference genes in meningioma qPCR experiments were further analyzed. After reducing the number of reference genes and increasing the number of samples both reference genes were considered one of the least stable genes. Bestkeeper-1 considered ACTB unsuitable as reference gene in meningiomas and their control tissues.

Because there is so few data available for gene expression of reference genes in meningiomas a large number of reference genes were screened. Using four randomly chosen meningiomas, the malignant meningioma cell line IOMM-Lee, pooled normal brain, cerebral meninges and dura mater was sufficient to determine expression levels of all reference genes as shown in Table 1. Because the four algorithms use different approaches for their rankings of the 32 reference genes, the ranking differed significantly making a selection of genes for further investigation difficult. Using the integration tool which weighs the ranking of each algorithm made the selection easier and more comprehensible. The six most stable reference genes according to the integration tool (PGK1, RPL37A, POP4, MRPL19, IPO8 and CASC3) were chosen for further analysis. Additionally PPIA and EIF2B1 were selected. PPIA was the highest ranked gene, which displayed high expression levels. EIF2B1 was the most stable gene with low expression levels. Because RPL30 is potentially co-regulated with RPL37A, it was not chosen, so the outcome of the result would not be affected.

For a more detailed analysis the remaining ten reference genes were analyzed using an increased number of samples (n_total _= 46 with n_normal _= 11 and n_meningioma _= 35) but a decreased number of software (NormFinder and Bestkeeper-1). NormFinder was chosen because of the model-based approach and the additional estimation of variation between normal and cancer tissue. In contrast Bestkeeper-1 employs a pair-wise correlation analysis and uses raw C_t _values whereas NormFinder uses transformed quantities. Also Bestkeeper-1 directly includes qPCR efficiency.

Both algorithms considered RPL37A as the most suitable reference gene for normalization in qPCR in meningiomas and their control tissue. The following ranking differed significantly especially for CASC3, IPO8 and EIF2B1. Bestkeeper-1 considered CASC3 as the most stable genes in meningiomas, but ranked CASC3 only in ninth place for normal control tissue. This led to a second place in the combined ranking due to the higher number of tumour samples. In contrast NormFinder ranked EIF2B1 highest for normal control tissue and only in sixth place in meningiomas. Because NormFinder weighs the two subgroups, normal tissue versus meningiomas, the ranking of the control tissue has more influence on the combined ranking. This is also demonstrated with IPO8 and conversely with RPL37A. NormFinder ranks RPL37A in meningiomas only in ninth place and in normal control tissue in second place. But after including the variation between those subgroups NormFinder displays RPL37A as the most stable gene for both subgroups.

Considering the results of the normalization of VEGFA against every single reference genes with significantly altered results for CASC3 and IPO8, NormFinder displays a more accurate ranking for meningiomas and their control tissue.

Some researchers recommend the use of multiple reference genes for calculating a normalization factor [23]. NormFinder also determines the best combination of two genes, when subgroups are included. For meningiomas and their normal control tissue the combination is RPL37A and EIF2B1.

## Conclusions

In conclusion, the results from the current study demonstrate that RPL37A is the most appropriate single reference gene for the normalization process of gene profiling studies in meningiomas and their normal control tissue arachnoidea, dura mater and normal brain. If a combination of reference genes is applicable RPL37A and EIF2B1 are most suitable. Additionally results from the current study indicate that widely used GAPDH and ACTB are both inappropriate reference genes for meningiomas.

## Competing interests

The authors declare that they have no competing interests.

## Authors' contributions

CP carried out cell cultivation and the Real-Time PCR studies, performed the statistical analysis and drafted the manuscript. MS supervised the study and reviewed the final version of the manuscript. FR conceived of the study, and participated in its design and coordination and helped to draft the manuscript. All authors read and approved the final manuscript.
